# Teleophthalmology adoption and perceived barriers among Colombian general practitioners: a cross-sectional study

**DOI:** 10.3389/fdgth.2026.1814108

**Published:** 2026-05-07

**Authors:** Valentina Loaiza-Guevara, Juliana P. Martinez-Rivera, Natalia Restrepo, Laura Quintero-Patino, Andrea Pinto-Arias, Luis Miguel Martinez, Wendy M. Rincón-Hernández, Alice Gaibor-Pazmiño, Juan S. Izquierdo-Condoy

**Affiliations:** 1 Facultad de Medicina, Universidad del Norte, Barranquilla, Colombia; 2 Facultad de Medicina, Fundación Universitaria Autónoma de las Américas, Pereira, Colombia; 3 Facultad de Medicina, Universidad de Caldas, Manizales, Colombia; 4 Facultad de Medicina, Universidad Autónoma de Bucaramanga, Bucaramanga, Colombia; 5 Facultad de Medicina, Universidad Militar Nueva Granada, Bogotá, Colombia; 6 Facultad de Medicina, Fundación Universitaria San Martin, Bogotá, Colombia; 7 One Health Research Group, Universidad de las Américas, Quito, Ecuador

**Keywords:** general practitioners, healthcare access, knowledge confidence, ophthalmology education, telemedicine, teleophthalmology

## Abstract

**Background:**

Telemedicine has improved access to healthcare, reduced costs, and minimized infection risks, particularly during the COVID-19 pandemic. Teleophthalmology may enhance access to eye care, but its adoption remains limited by barriers such as inadequate training and insufficient infrastructure.

**Objectives:**

To assess perceptions, self-reported ophthalmologic confidence, and perceived barriers related to teleophthalmology among Colombian general practitioners.

**Methods:**

We conducted a cross-sectional online survey between February and May 2024 using a 31-question questionnaire covering demographics, prior training, telemedicine experiences, ophthalmologic confidence, and teleophthalmology perceptions. Composite Likert-based scores were categorized into three levels. Internal consistency was assessed using Cronbach's alpha, and associations were evaluated using chi-square tests and multivariable binary logistic regression.

**Results:**

Among 489 participants, most were women (62.2%), aged 21–30 years (70.1%), and had 0–5 years of professional experience (65.6%). Undergraduate telemedicine training was uncommon (9.0%), and prior teleophthalmology experience was rare (1.8%). Confidence in ophthalmologic knowledge was mostly moderate (45.6%), whereas 57.3% reported positive perceptions of teleophthalmology. In adjusted analyses, greater undergraduate ophthalmology training was independently associated with high ophthalmologic confidence, with a dose-response pattern, including 51–100 h (aOR: 8.82, 95% CI: 2.88–27.08) and >100 h (aOR: 14.71, 95% CI: 3.86–55.97). Prior experience providing telemedicine consultations was also associated with high confidence (aOR: 1.92, 95% CI: 1.16–3.18). Moderate ophthalmologic confidence was independently associated with positive teleophthalmology perception (aOR: 2.46, 95% CI: 1.60–3.80).

**Conclusions:**

Colombian general practitioners showed generally favorable perceptions of teleophthalmology despite limited prior exposure and persistent concerns regarding privacy, workload, job security, training, and implementation readiness. Strengthening ophthalmology and telemedicine training, together with digital readiness, may support teleophthalmology integration into routine practice.

## 1 Introduction

Telemedicine, defined as the delivery of healthcare, education, and information services through remote technologies, has revolutionized healthcare access and delivery. Initially reliant on rudimentary telephone and video technology in the 1970s, the advent of the Internet, wireless networks, and mobile technologies has transformed telemedicine into an efficient, accessible, and widely adopted solution for overcoming barriers to healthcare delivery (1, 2). Telemedicine has proven especially valuable in addressing healthcare disparities by minimizing costs, reducing travel time, and lowering infection risks—benefits that became particularly evident during the COVID-19 pandemic, which accelerated its adoption worldwide (3, 4). As a result, telemedicine has established itself as a cornerstone of modern healthcare, enabling equitable access to care across urban and rural populations (5).

Within this framework, teleophthalmology has emerged as a critical branch of telemedicine, addressing the growing global demand for ophthalmic care (6). This field has demonstrated success in processes such as the screening, diagnosis, and follow-up of ophthalmologic conditions, including diabetic retinopathy, glaucoma, retinopathy of prematurity, macular degeneration, and other eye diseases (7–9). Technological advances have enabled teleophthalmology to become a widely accepted clinical solution, improving access to eye care services, particularly in underserved areas. However, despite its potential, important challenges remain. Barriers such as insufficient training, fragmented organizational structures, and limited communication among stakeholders have hindered the smooth integration of teleophthalmology into primary healthcare workflows (10, 11). In addition, inequalities in access to teleophthalmology services persist, particularly among socioeconomically disadvantaged populations, reinforcing a “digital divide” that demands urgent attention. In this context, general practitioners (GPs), as primary care providers, play a fundamental role in overcoming these barriers and ensuring the successful adoption of teleophthalmology (12, 13).

In Colombia, where geographic, economic, and systemic factors significantly restrict access to specialized ophthalmologic care, teleophthalmology presents an innovative solution to bridge these gaps. Despite its potential, there remains a lack of in-depth understanding of the knowledge, perceptions, and attitudes of GPs toward teleophthalmology—insights that are essential for its effective implementation and integration into the healthcare system.

This study aimed to assess the perceptions, self-reported confidence, and perceived barriers related to teleophthalmology among general practitioners in Colombia.

## 2 Materials and methods

### 2.1 Study design

This cross-sectional, observational study was conducted using a self-administered online survey targeting general practitioners in Colombia between February and May 2024.

### 2.2 Setting and population

Colombia, a Latin American country, is bordered by the Pacific Ocean and shares borders with Ecuador, Peru, Brazil, Venezuela, and Panama, regions that have significant cultural influences (14). According to the Colombian Ministry of Health, approximately 126,279 physicians were practicing in 2022, of whom 94,892 were general practitioners (75.1%) (15).

The study population consisted of general practitioners residing in Colombia, defined as individuals with a university degree in medicine who had not pursued or completed postgraduate specialization studies.

### 2.3 Sample

Based on the 2022 estimate of 94,892 general practitioners (15), the sample size was calculated using a 95% confidence level, a 5% margin of error, and a 50% response probability. The minimum required sample was 383 participants, determined using a standard formula for survey populations (16):

$$n=\frac{\left(N.Z^{2}).(\;p.q\right)}{d^{2}.(N-1)+Z^{2}.p.q}$$

A non-probabilistic convenience sampling method was employed using the “Google Forms” platform. Participation was voluntary, and only responses from those who provided informed consent were included.

### 2.4 Questionnaire development and measurement

The study used a 31-question, self-administered online questionnaire developed by the research team. The instrument underwent a two-step validation process. First, two experts in telemedicine and ophthalmology reviewed the questionnaire for clarity, relevance, and content adequacy. Their feedback was incorporated into a revised version, which was then pilot tested in 30 general practitioners to evaluate comprehension, wording, and response flow. Based on this process, the final Spanish-language questionnaire was structured into six sections:

- Informed consent
- Sociodemographic data (3 questions)
- Previous training (2 questions)
- Previous experiences and perceptions related to telemedicine (5 questions)
- Confidence towards knowledge in different clinical scenarios in ophthalmology (10 questions)
- Perceptions towards teleophthalmology (10 questions)

In addition, internal consistency of the confidence and perception scales was assessed using Cronbach's alpha (*α*).

### 2.5 Variables

The questionnaire collected various variables, including demographic data (e.g., gender, age, and work experience). Additionally, it gathered information on prior training, specifically telemedicine training and the number of hours dedicated to ophthalmology during undergraduate studies.

Perceptions and experiences related to telemedicine were assessed from both patient and physician perspectives, based on prior exposure to telemedicine services. Confidence in ophthalmology knowledge was evaluated across ten of the most common clinical scenarios encountered in general medical practice (17). Responses regarding confidence levels were measured using a 5-point Likert scale: *not at all confident, slightly confident, somewhat confident, confident, and very confident.* To determine overall confidence in ophthalmology knowledge, numerical values were assigned to each response: *1* *=* *not at all confident, 2* *=* *slightly confident, 3* *=* *somewhat confident, 4* *=* *confident, and 5* *=* *very confident.* The mean confidence score was then calculated and categorized into three levels: *low confidence* (1.00–2.49), *moderate confidence* (2.50–3.49), and *high confidence* (3.50–5.00).

General practitioners' perceptions of teleophthalmology were assessed through ten potential scenarios exploring its perceived positive or negative effects. Responses were recorded using a 5-point Likert scale: *strongly disagree, disagree, somewhat agree, agree, and strongly agree.* To determine the overall perception of teleophthalmology, numerical values were assigned to each response: *1* *=* *strongly disagree, 2* *=* *disagree, 3* *=* *somewhat agree, 4* *=* *agree, and 5* *=* *strongly agree.* The mean perception score was then calculated and categorized into three levels: *negative perception* (1.00–2.49), *neutral perception* (2.50–3.49), and *positive perception* (3.50–5.00).

### 2.6 Data collection and management

Data collection was conducted using the online platform Google Forms, with participants accessing the survey through a unique link disseminated via social media channels, including Instagram and WhatsApp. The survey preamble provided an overview of the study objectives, reinforced the importance of confidentiality, and included a request for informed consent. Participants were required to agree to an electronic Participation Agreement before proceeding, ensuring anonymity throughout the process.

To maintain the highest standards of data integrity, responses underwent a meticulous review process. This included checking for inconsistencies such as implausible age ranges, responses where all options were selected indiscriminately, or duplicate submissions. Surveys that only contained demographic data were also excluded. Out of 567 initial responses, 78 did not meet the inclusion criteria and were removed following this rigorous quality control process. Ultimately, 489 valid responses were retained for inclusion in the final analysis.

### 2.7 Ethical statement

This study adhered to the ethical principles of the Declaration of Helsinki and was approved by the Ethics Committee of Red Medica Vital S.A. (approval code: 3112-0824; approval date: 16 January 2024). No personal, identifiable, high-risk, or sensitive information was collected. All participants provided electronic informed consent before completing the survey.

### 2.8 Statistical analysis

Descriptive statistics were used to summarize categorical variables as frequencies and percentages. For the ophthalmologic confidence and teleophthalmology perception domains, individual Likert items were coded from 1 to 5 and averaged to generate composite scores, as this is an accepted approach when items measure a common construct and demonstrate adequate internal consistency. Composite scores were categorized as low/negative (1.00–2.49), moderate/neutral (2.50–3.49), and high/positive (3.50–5.00), reflecting the conceptual anchors of the 5-point Likert scale. Internal consistency was assessed using Cronbach's alpha for both scales, with values ≥0.70 considered acceptable.

Chi-square tests were used to explore associations between demographic characteristics, prior training, and telemedicine-related experiences with confidence in ophthalmologic knowledge. Similarly, associations between demographic characteristics, training, telemedicine-related experiences, overall ophthalmologic confidence, and general perceptions toward teleophthalmology were examined.

To account for potential confounding, two multivariable binary logistic regression models were performed. The first model evaluated independent predictors of high ophthalmologic confidence, defined as high confidence vs. low or moderate confidence. The second model evaluated independent predictors of positive teleophthalmology perception, defined as positive perception vs. neutral or negative perception. Variables were selected based on conceptual relevance and exploratory bivariate findings. Adjusted odds ratios (aORs) with 95% confidence intervals (CIs) were reported. Statistical significance was set at *p* < 0.05. All analyses were performed using IBM SPSS Statistics for Windows, version 26.0 (IBM Corp., Chicago, IL, USA).

## 3 Results

A total of 489 Colombian general practitioners were included. Most respondents were women (62.2%), aged 21–30 years (70.1%) and had 0–5 years of professional experience (65.6%). Undergraduate telemedicine training was uncommon, and prior direct exposure to teleophthalmology was minimal (Table 1). The composite scales showed adequate internal consistency. Cronbach's alpha was 0.90 for the ophthalmologic confidence scale, indicating excellent reliability, and 0.81 for the teleophthalmology perception scale, indicating good reliability.

**Table 1:** Demographic characteristics, prior training, and telemedicine-related experiences among Colombian general practitioners (*n* = 489).

Variable	Categories	*n*	%
Demographic characteristics			
Age (years)	21 to 30	343	70.1
	31 to 40	125	25.6
	Over 40	21	4.3
Sex	Female	304	62.2
	Male	185	37.8
Professional Experience (years)	0 to 5	321	65.6
	6 to 10	126	25.8
	More than 10	42	8.6
Previous training			
Telemedicine training in undergrad	No	445	91.0
	Yes	44	9.0
Ophthalmology training hours during undergraduate	Less than 10 h	71	14.5
	10–25 h	140	28.6
	26–50 h	172	35.2
	51–100 h	82	16.8
	More than 100 h	24	4.9
Telemedicine-related experiences			
Previous experience as a telemedicine patient	No	199	40.7
	Yes	290	59.3
Previous experience as a healthcare professional providing telemedicine consultations	No	196	40.1
	Yes	293	59.9
Previous experience as a healthcare professional consulting another professional via telemedicine	No	257	52.6
	Yes	232	47.4
Do you have prior experience using telemedicine in ophthalmology?	No	351	71.8
	Yes	9	1.8
	I did not know telemedicine could be used in ophthalmology	129	26.4
Would you be willing to consult with an ophthalmologist through a virtual appointment?	No	98	20
	Yes	391	80

Values are presented as frequencies (n) and percentages (%).

### 3.1 Physicians' confidence in their ophthalmologic knowledge

When assessing physicians' confidence in their knowledge of common ophthalmologic conditions, low confidence was predominant in several scenarios: managing refractive disorders such as myopia, hyperopia, and astigmatism (30.1%); diagnosing and treating blepharitis (30.7%); evaluating visual symptoms with no apparent cause (42.9%); managing early-stage glaucoma (39.5%); and treating cataracts (33.1%).

**Figure 1:**
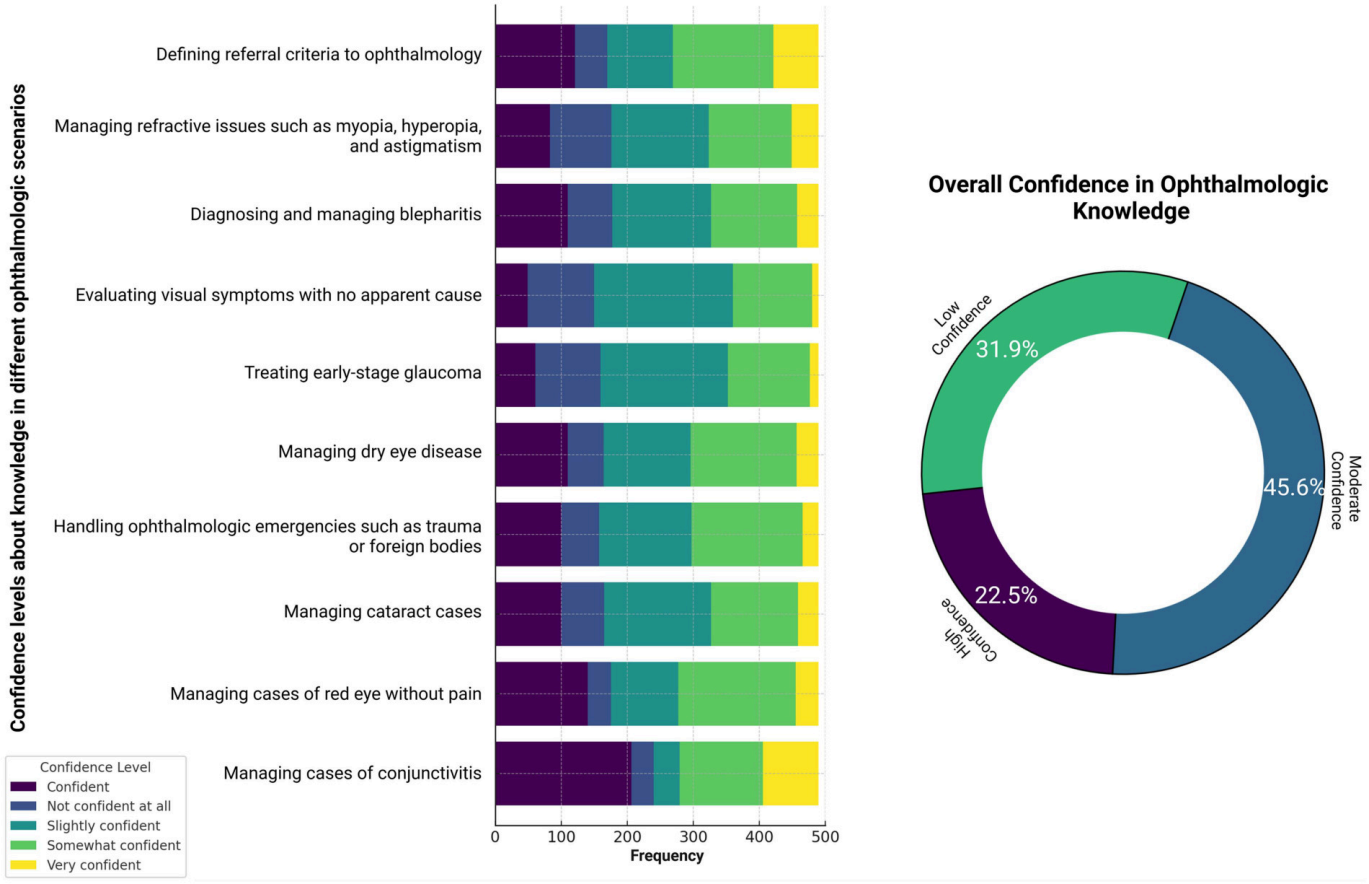
Distribution of self-reported confidence levels (low, moderate, and high) among Colombian general practitioners across 10 common ophthalmologic clinical scenarios and for the overall ophthalmologic confidence score (*n* = 489). Confidence categories were derived from the mean composite Likert score.

Conversely, moderate confidence was the most frequent response for managing red eye without pain (36.4%), handling ophthalmologic emergencies (34.4%), and treating dry eye disease (32.7%). Notably, the only scenario where participants expressed predominantly high confidence was in managing conjunctivitis (42.1%) (Figure 1). When assessing overall confidence in ophthalmology, a moderate confidence level was the most common (45.6%) (Figure 1).

### 3.2 Factors associated with confidence in ophthalmologic knowledge

In bivariate analyses, confidence in ophthalmologic knowledge was significantly associated with undergraduate telemedicine training, ophthalmology training hours during undergraduate education, prior experience providing telemedicine consultations, and prior professional-to-professional telemedicine consultation (Table 2). Higher levels of undergraduate ophthalmology training were associated with a greater proportion of high confidence, with the highest value observed among physicians reporting more than 100 h of training (45.8%). High confidence was also more frequent among physicians who had received telemedicine training during undergraduate studies (36.4% vs. 21.1%, *p* = 0.030) and among those with prior experience providing telemedicine consultations (27.0% vs. 15.8%, *p* = 0.015) (Table 2).

**Table 2:** Factors associated with the level of confidence in general ophthalmologic knowledge Among Colombian general practitioners (*n* = 489).

		Confidence in ophthalmologic knowledge						
Variable	Categories	Low confidence		Moderate confidence		High confidence		
		*n*	%	*n*	%	*n*	%	*p*-value
Age (years)	21 to 30	101	29.4	158	46.1	84	24.5	0.216
	31 to 40	45	36.0	56	44.8	24	19.2	
	Over 40	10	47.6	9	42.9	2	9.5	
Sex	Female	103	33.9	136	44.7	65	21.4	0.458
	Male	53	28.6	87	47	45	24.3	
Professional Experience (years)	0 to 5	97	30.2	148	46.1	76	23.7	0.792
	6 to 10	43	34.1	57	45.2	26	20.6	
	More than 10	16	38.1	18	42.9	8	19.0	
Telemedicine training in undergrad	No	148	33.3	203	45.6	94	21.1	0.030
	Yes	8	18.2	20	45.5	16	36.4	
Ophthalmology training hours during undergraduate (hours)	Less than 10	30	42.3	37	52.1	4	5.6	0.001
	10–25	59	42.1	57	40.7	24	17.1	
	26–50	46	26.7	84	48.8	42	24.4	
	51–100	16	19.5	37	45.1	29	35.4	
	More than 100	5	20.8	8	33.3	11	45.8	
Previous experience as a telemedicine patient	No	69	34.7	92	46.2	38	19.1	0.278
	Yes	87	30.0	131	45.2	72	24.8	
Previous experience as a healthcare professional providing telemedicine consultations	No	69	35.2	96	49.0	31	15.8	0.015
	Yes	87	29.7	127	43.3	79	27.0	
Previous experience as a healthcare professional consulting another professional via telemedicine	No	96	37.4	102	39.7	59	23.0	0.010
	Yes	60	25.9	121	52.2	51	22.0	
Do you have prior experience using telemedicine in ophthalmology?	No	111	31.6	158	45.0	82	23.4	0.558
	Yes	4	44.4	2	22.2	3	33.3	
	I did not know telemedicine could be used in ophthalmology	41	31.8	63	48.8	25	19.4	
Would you be willing to consult with an ophthalmologist through a virtual appointment?	No	29	29.6	47	48.0	22	22.4	0.838
	Yes	127	32.5	176	45.0	88	22.5	

Values are presented as frequencies (n) and percentages (%). Associations were assessed using the chi-square test. Statistical significance was set at *p* < 0.05.

In the adjusted model, greater undergraduate ophthalmology training remained independently associated with high ophthalmologic confidence. Compared with physicians reporting fewer than 10 h of undergraduate ophthalmology training, those with 10–25 h (aOR: 3.43, 95% CI: 1.13–10.42), 26–50 h (aOR: 5.04, 95% CI: 1.72–14.79), 51–100 h (aOR: 8.82, 95% CI: 2.88–27.08), and more than 100 h (aOR: 14.71, 95% CI: 3.86–55.97) had progressively higher odds of reporting high confidence in ophthalmologic knowledge. Prior experience providing telemedicine consultations was also independently associated with high confidence (aOR: 1.92, 95% CI: 1.16–3.18) (Table 3).

**Table 3:** Multivariable logistic regression for factors associated with high ophthalmologic confidence among Colombian general practitioners (*n* = 489).

Variable	Categories	aOR	95% CI	*p*-value
Age (years)	21–30	*Ref.*	—	—
	31–40	0.67	0.39–1.14	0.141
	>40	0.22	0.05–1.00	0.050
Sex	Female	*Ref.*	—	—
	Male	1.10	0.70–1.75	0.680
Telemedicine training in undergrad	No	*Ref.*	—	—
	Yes	1.77	0.87–3.59	0.113
Ophthalmology training during undergraduate (hours)	<10	*Ref.*	—	—
	10–25	3.43	1.13–10.42	0.030
	26–50	5.04	1.72–14.79	0.003
	51–100	8.82	2.88–27.08	<0.001
	>100	14.71	3.86–55.97	<0.001
Prior experience providing telemedicine consultations	No	*Ref.*	—	—
	Yes	1.92	1.16–3.18	0.012
Prior professional-to-professional telemedicine consultation	No	*Ref.*	—	—
	Yes	0.73	0.45–1.17	0.194

Adjusted odds ratios (aORs) and 95% confidence intervals (CIs) were estimated using multivariable binary logistic regression. The outcome was defined as high ophthalmologic confidence vs. low/moderate confidence.

### 3.3 Perceptions of teleophthalmology

**Figure 2:**
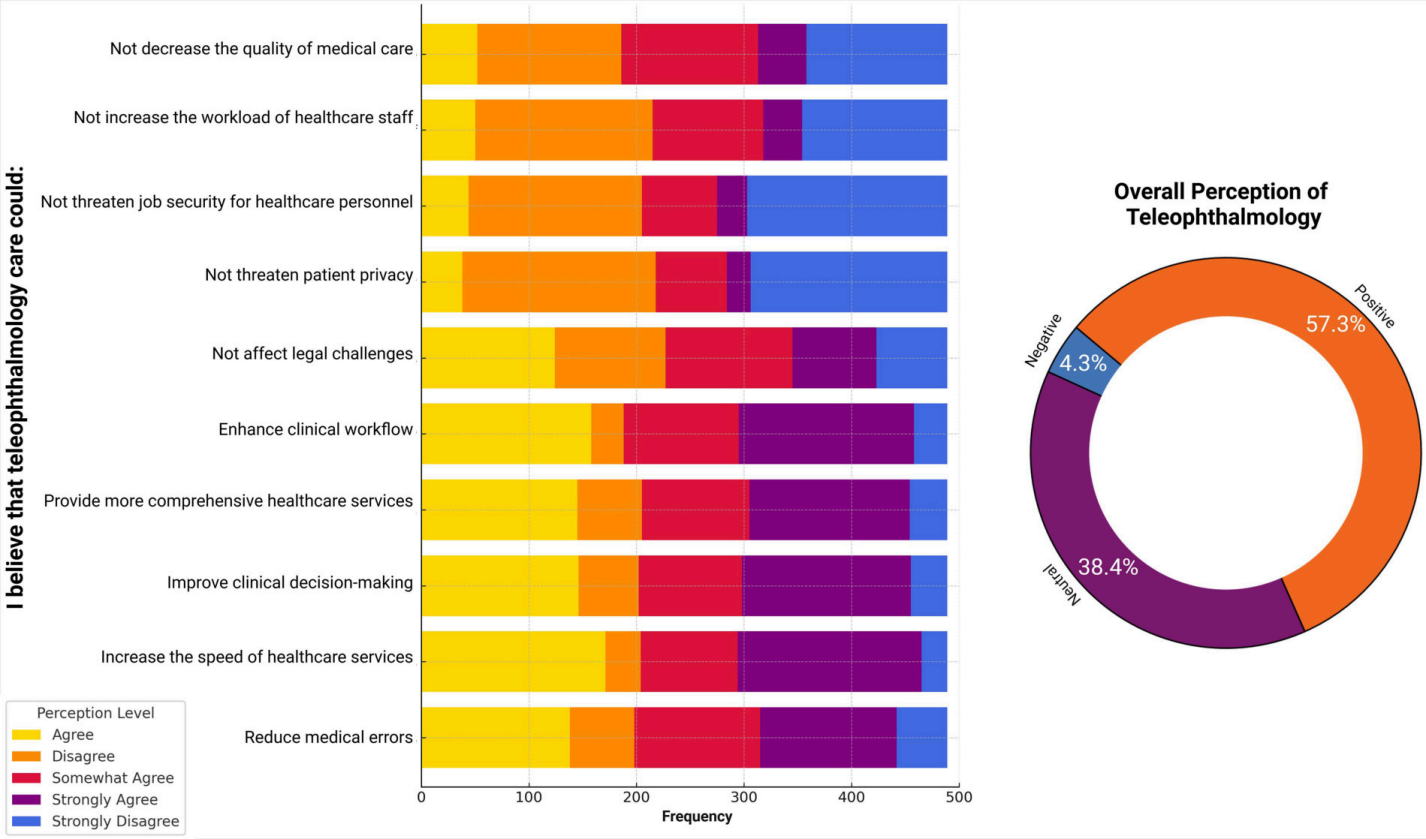
Distribution of item-level perceptions toward teleophthalmology and overall perception categories (negative, neutral, and positive) among Colombian general practitioners (*n* = 489). Perception categories were derived from the mean composite Likert score.

Participants expressed notable concern regarding patient privacy (37.4%) and the potential impact of teleophthalmology on job security (38.0%). At the same time, many disagreed that teleophthalmology would increase workload or reduce the quality of care (33.7%). On the other hand, participants strongly agreed that teleophthalmology could improve clinical workflow (33.3%), provide more comprehensive healthcare services (30.5%), and enhance clinical decision-making (32.1%). There was also a predominant agreement that teleophthalmology could speed up medical care services (35.0%) and reduce medical errors (28.2%) (Figure 2). The overall perception of teleophthalmology was positive in 57.3% of participants (Figure 2).

### 3.4 Factors associated with perceptions of teleophthalmology

In bivariate analyses, positive teleophthalmology perception was significantly associated with willingness to consult an ophthalmologist via telemedicine and with overall ophthalmologic confidence, whereas demographic characteristics, undergraduate training, and prior telemedicine experiences were not significantly associated with the outcome (Table 4). Positive perception was particularly frequent among participants willing to consult virtually with an ophthalmologist (67.8%; *p* < 0.001) and among those with moderate ophthalmologic confidence (66.8%; *p* < 0.001) (Table 4).

**Table 4:** Factors associated with the general perception of teleophthalmology Among Colombian general practitioners (*n* = 489).

		Perception of Teleophthalmology						
Variable	Categories	Negative			Neutral		Positive	
		*n*	%	*n*	%	*n*	%	*p*-value
Age (years)	21 to 30	15	4.4	119	34.7	209	60.9	0.07
	31 to 40	6	4.8	57	45.6	62	49.6	
	Over 40	0	0.0	12	57.1	9	42.9	
Sex	Female	14	4.6	108	35.5	182	59.9	0.233
	Male	7	3.8	80	43.2	98	53	
Professional Experience (years)	0 to 5	15	4.7	111	34.6	195	60.7	0.12
	6 to 10	4	3.2	55	43.7	67	53.2	
	More than 10	2	4.8	22	52.4	18	42.9	
Telemedicine training in undergrad	Yes	2	4.5	16	36.4	26	59.1	0.956
	No	19	4.3	172	38.7	254	57.1	
Ophthalmology training hours during undergraduate (hours)	Less than 10	4	5.6	28	39.4	39	54.9	0.787
	10–25	8	5.7	56	40.0	76	54.3	
	26–50	5	2.9	59	34.3	108	62.8	
	51–100	3	3.7	34	41.5	45	54.9	
	More than 100	1	4.2	11	45.8	12	50.0	
Previous experience as a telemedicine patient	No	7	3.5	78	39.2	114	57.3	0.771
	Yes	14	4.8	110	37.9	166	57.2	
Previous experience as a healthcare professional providing telemedicine consultations	No	8	4.1	66	33.7	122	62.2	0.183
	Yes	13	4.4	122	41.6	158	53.9	
Previous experience as a healthcare professional consulting another professional via telemedicine	No	14	5.4	92	35.8	151	58.8	0.237
	Yes	7	3.0	96	41.4	129	55.6	
Do you have prior experience using telemedicine in ophthalmology?	No	11	3.1	142	40.5	198	56.4	0.143
	Yes	0	0.0	4	44.4	5	55.6	
	I did not know telemedicine could be used in ophthalmology	10	7.8	42	32.6	77	59.7	
Would you be willing to consult with an ophthalmologist through a virtual appointment?	No	15	15.3	68	69.4	15	15.3	0.001
	Yes	6	1.5	120	30.7	265	67.8	
Overall confidence in ophthalmologic knowledge	High Confidence	2	1.8	47	42.7	61	55.5	0.001
	Moderate Confidence	8	3.6	66	29.6	149	66.8	
	Low Confidence	11	7.1	75	48.1	70	44.9	

Values are presented as frequencies (n) and percentages (%). Associations were assessed using the chi-square test. Statistical significance was set at *p* < 0.05.

In the multivariable binary logistic regression model, moderate ophthalmologic confidence remained independently associated with positive teleophthalmology perception when compared with low confidence (aOR: 2.46, 95% CI: 1.60–3.80). High confidence showed a positive but non-significant association with positive teleophthalmology perception (aOR: 1.53, 95% CI: 0.90–2.59). In addition, age, sex, undergraduate telemedicine training, undergraduate ophthalmology training hours, and prior experience providing telemedicine consultations were not independently associated with the outcome (Table 5).

**Table 5:** Multivariable logistic regression for factors associated with positive teleophthalmology perception among Colombian general practitioners (*n* = 489).

Variable	Categories	aOR	95% CI	*p*-value
Age (years)	21–30	*Ref.*	—	—
	31–40	0.70	0.46–1.08	0.105
	>40	0.57	0.22–1.45	0.238
Sex	Female	*Ref.*	—	—
	Male	0.76	0.52–1.12	0.171
Telemedicine training in undergrad	No	*Ref.*	—	—
	Yes	1.11	0.58–2.14	0.758
Ophthalmology training during undergraduate (hours)	<10	*Ref.*	—	—
	10–25	1.14	0.63–2.08	0.660
	26–50	1.42	0.79–2.55	0.247
	51–100	1.05	0.53–2.07	0.886
	>100	0.99	0.37–2.66	0.988
Ophthalmologic confidence	Low	*Ref.*	—	—
	Moderate	2.46	1.60–3.80	<0.001
	High	1.53	0.90–2.59	0.114
Prior experience providing telemedicine consultations	No	*Ref.*	—	—
	Yes	0.74	0.50–1.10	0.138

Adjusted odds ratios (aORs) and 95% confidence intervals (CIs) were estimated using multivariable binary logistic regression. The outcome was defined as positive teleophthalmology perception vs. neutral/negative perception.

## 4 Discussion

To our knowledge, this is the first study to evaluate Colombian general practitioners' perceptions of teleophthalmology. Our findings suggest that teleophthalmology readiness among Colombian general practitioners is mainly shaped by workforce profile, ophthalmology training, and perceived barriers to implementation. Our sample was predominantly female (62.2%), a finding consistent with national and regional trends showing a growing predominance of female physicians and women medical students in recent years (18–21). In addition, the study sample was composed predominantly of physicians in the early stages of their professional careers, which may have contributed to the generally favorable attitudes toward digital care. Furthermore, self-reported confidence in ophthalmologic knowledge was mostly low to moderate and showed a clear independent association with the amount of undergraduate ophthalmology training received. On the other hand, although direct experience with teleophthalmology was infrequent, overall perceptions were predominantly positive, suggesting openness to its adoption despite limited practical familiarity.

Although previous ophthalmology training showed diverse trends, undergraduate training in telemedicine was found to be almost nonexistent among participants, a pattern similar to that reported in previous studies from different regions (22, 23). In addition, prior experiences with telemedicine were relevant from the patient's perspective, but considerably less frequent in the role of healthcare provider. These limitations were even more pronounced in teleophthalmology, as only 1.8% of the sample reported previous access to these services. Likewise, likely reflecting differences in undergraduate training, our results showed that general practitioners' confidence in diagnosing and managing ophthalmologic diseases was generally low, particularly for conditions such as blepharitis, early-stage glaucoma, and cataract, whereas they reported greater confidence in managing a more common condition such as conjunctivitis. This highlights the insufficiency of ophthalmology training in general medical education programs, in line with previous studies that have identified gaps in ophthalmology teaching within undergraduate curricula (24–26).

A key finding was that confidence in ophthalmologic knowledge was independently associated with the number of hours of ophthalmology training received during undergraduate education. Compared with those who had received fewer than 10 h of training, all categories of greater exposure were associated with progressively higher odds of reporting high confidence, suggesting a directly proportional relationship. This finding reinforces the importance of strengthening undergraduate ophthalmology education, particularly in settings such as Colombia, where access to specialists may be uneven and general practitioners often represent the first point of contact for patients with ocular complaints (27–29).

Furthermore, prior experience providing telemedicine consultations in general remained independently associated with greater teleophthalmology confidence. Although this does not imply a causal effect, it suggests that familiarity with remote care delivery may enhance physicians' confidence in clinical assessment and decision-making within virtual environments. This may reflect broader gains in digital clinical communication, triage, and remote case management acquired through telemedicine practice (30, 31).

Overall, physicians' perceptions of teleophthalmology were predominantly positive, at 57.3%. In this context, the barriers identified in this study can be broadly categorized into organizational, technological, and educational domains. Our findings showed that the most relevant concerns were related to privacy, workload, job security, and integration into clinical workflows. Broader technological barriers, such as connectivity limitations and restricted access to diagnostic equipment, have also been described in the literature and are likely to be relevant for implementation in resource-limited settings. In addition, educational barriers were reflected in the limited training in both ophthalmology and telemedicine, which may reduce confidence and hinder adoption. This framework helps contextualize the challenges associated with teleophthalmology implementation and is consistent with previous telemedicine research describing infrastructure gaps and concerns about depersonalized care as important barriers to its acceptance (32, 33).

One of the main advantages of teleophthalmology is its potential to improve access to ophthalmologic care in regions with shortages of specialists, such as Colombia, where significant inequalities exist in the distribution of health services (34). From a systems perspective, these findings suggest that teleophthalmology could help reduce disparities in access to specialized eye care, especially in underserved regions. However, its implementation requires coordinated efforts in infrastructure development, workforce training, and regulatory support. Without addressing these factors, large-scale adoption may remain limited. Evidence has shown that teleophthalmology can reduce the burden of unnecessary in-person consultations, improve health system efficiency, and provide more equitable access to eye care for rural and underserved communities (35–37). Nevertheless, major technological investments, including reliable internet access and adequate imaging systems, remain a challenge in resource-limited settings (1, 32, 38).

Additionally, in the adjusted model, moderate ophthalmologic confidence was independently associated with a positive perception of teleophthalmology, whereas high confidence showed a similar direction of association but did not reach statistical significance. This pattern may suggest that a minimum threshold of clinical self-efficacy, rather than very high confidence alone, is relevant for openness toward adoption of teleophthalmology. In other words, physicians who feel sufficiently prepared to assess common ophthalmologic conditions may be more willing to perceive teleophthalmology as a useful extension of care.

It is important to note that our findings underscore healthcare provider training in teleophthalmology as a crucial factor for its success. Previous studies have highlighted that inadequate communication and insufficient training may generate resistance among medical staff, negatively affecting collaboration and the effectiveness of telemedicine programs (39, 40). These findings support the need for targeted strategies, including the incorporation of telemedicine and, where possible, teleophthalmology into undergraduate medical curricula, the development of structured continuing education programs, and the implementation of policies that support digital health integration. Strengthening these areas could facilitate adoption and improve the effectiveness of teleophthalmology in clinical practice.

Finally, patient experience should be considered in the adoption of teleophthalmology. Studies have shown that patients, particularly those living in rural areas, highly value the possibility of receiving ophthalmologic care without having to travel long distances. This improves adherence to medical follow-up and facilitates the early detection of ocular conditions such as diabetic retinopathy (41, 42).

### 4.1 Limitations

This study has several limitations. First, the sample was predominantly composed of young general practitioners with limited professional experience, which may restrict the generalizability of the findings to the broader population of Colombian physicians. This demographic concentration may have influenced the generally positive perceptions observed, as early-career physicians may be more familiar with digital technologies and may hold more favorable attitudes toward telemedicine. In addition, recruitment was conducted through an online self-administered survey distributed via social media, which may have introduced selection bias by favoring participation among physicians with greater digital engagement or a stronger interest in telemedicine. Because the survey link was disseminated through open digital channels, it was not possible to estimate a response rate.

The study also relied on self-reported measures, which may be susceptible to response bias and may not fully reflect actual clinical practice. In particular, ophthalmologic preparedness was assessed through self-reported confidence rather than objective clinical evaluations or case-based performance measures. Future research incorporating validated competency frameworks or practical assessments would provide a more precise estimate of physicians' preparedness to diagnose and manage ophthalmologic conditions.

Although the confidence and perception scales showed good internal consistency, additional psychometric evaluation would further strengthen the instrument for future use. Specifically, future studies should assess construct validity to determine whether the questionnaire items cluster as expected within the confidence and perception domains, ideally through exploratory and confirmatory factor analyses. Additional evaluation of test–retest reliability and convergent validity would also support the robustness of the instrument. Moreover, some subgroup categories were relatively small, particularly physicians with prior teleophthalmology experience and older age groups. For example, only 9 participants reported prior teleophthalmology experience, and only 21 were older than 40 years; similarly, the subgroup reporting more than 100 h of undergraduate ophthalmology training was also small. These limited subgroup sizes may have reduced statistical power and precision, yielding less stable estimates and wider confidence intervals in some comparisons. Future studies should address this limitation by recruiting larger and more heterogeneous samples, ideally with stratified sampling or targeted recruitment of underrepresented physician subgroups.

Finally, the study did not assess implementation feasibility in depth, including logistical, financial, or institutional barriers to large-scale adoption. Because the study was conducted in a single country, the external validity of the findings may also be limited, as healthcare infrastructure, telemedicine regulation, and medical education frameworks vary across settings.

## 5 Conclusions

To our knowledge, this study represents the first exploration of teleophthalmology perceptions among general practitioners in Colombia and primarily reflects the views of early-career physicians. Confidence in ophthalmologic knowledge appears to be an important factor in teleophthalmology readiness. In particular, greater undergraduate ophthalmology training and prior experience providing telemedicine consultations were independently associated with higher ophthalmologic confidence, while moderate ophthalmologic confidence was independently associated with a positive perception of teleophthalmology.

Although perceptions of teleophthalmology were generally favorable, major concerns remained regarding privacy, workload, job security, training, and implementation readiness. Given the potential of teleophthalmology to improve access to vision care in underserved settings, academic institutions and healthcare systems should strengthen ophthalmology and telemedicine training, improve digital readiness, and support structured integration into routine clinical practice. Future studies should include more heterogeneous physician groups and implementation-focused designs to better inform policy and service development in Colombia.

## Data Availability

The original contributions presented in the study are included in the article/Supplementary Material, further inquiries can be directed to the corresponding author.
